# Notes and News

**Published:** 1895-12-21

**Authors:** 


### NOTES AND NEWS.

(COLLECTED AND COMMUNICATED.)

COLONEL W. ROE, of the Indian Medical Service, has been appointed honorary secretary to the association formed to promote a Pasteur Institute for India.

IT is reported that the Local Government Board authorities have resolved to insist on the immediate provision of two additional fever hospitals for the metropolis, to accommodate five hundred patients each.

IT has afforded the Editor of THE HOSPITAL much gratification to receive the following letter from the Secretary-Director of the National Hospital for the Paralysed and Epileptic:

Among the rules for the constitution of the Charity is one which empowers the Board of Management to add to the list of Governors anyone who, in their opinion, has rendered the Charity an important service.

I have the pleasure and satisfaction to inform you that at their meeting yesterday the Board, with a due sense of the assistance you have been the means of affording to this, among the other hospitals of London, unanimously elected you to be an Honorary Life Governor, subject to its being agreeable to you to accept this position.

I hope to be favoured with the necessary permission to place your name upon the Register.

A REPORT has been presented by a committee which had been appointed to inquire into the alleged excessive infantile mortality among the Jews in London. We cannot look upon their conclusions as very convincing. It is quite clear that, owing to the very large proportion of Jewish immigrants who on arriving in London are between twenty and forty years of age and quickly become parents, the age distribution is greatly disturbed, so that the statement that the infantile mortality is very heavy in proportion to the total mortality may not mean much; but it would have been much more satisfactory to have been informed what is the annual mortality at each age period. It would then have been possible to make a proper comparison between the Jews and the other peoples among whom they dwell. As the report stands, no such information is given.

SCIENCE is frequently claiming its victims to research. One of the most recent is Dr. Wörtoff, Professor of Bacteriology in Moscow. He died through infecting himself with a virulent culture with which he was experimenting.

THE Admiralty have decided to establish a convalescent hospital for the use of cadets undergoing training on Her Majesty's ship *Britannia* in Dartmouth Harbour, so that patients may be treated ashore instead of in the ship. A suitable site has been acquired at Kingswear.

THE School Committee of the Brentwood Schools have decided that the children shall not be submitted to treatment by anti-toxin, as they regard such treatment in the experimental stage. Dr. Wallis, the medical officer to the Brentwood Schools, had stated that he should adopt the treatment when he regarded it as necessary, hence the decision of the committee.

FOR some time the need of further accommodation at the Edinburgh Royal Infirmary has been patent. At a recent meeting of the managers it was announced that £30,000 towards the estimated expenditure of £100,000 was available. It was decided to proceed with the erection of laundry premises at once, and to leave the choice as to the special medical department, next to be proceeded with, to the consideration of the medical and surgical staff.

WE are informed that the metric system will be introduced into the new edition of the British Pharmacopœia. This is an important advance in the direction of the general adoption of metric weights and measures in this country, which step, it will be remembered, was recently recommended by a Select Committee of the House of Commons. The metric system of weights and measures is to be legalised for all purposes and taught in elementary schools.

THE annual meeting of the Livingstone College was held recently, upon which occasion the new buildings were opened. The object of the college is to give intending missionaries for foreign parts a useful medical training of an elementary nature. Clinical work is done at the West Ham and at the Poplar Hospital for Accidents, and other tuition is in the hands of the principal, Dr. Charles Harford-Battersby, and Mr. McAdam Eccles.

THE *Daily Chronicle* has collected £3,000, and even more, for an isolation block in connection with the Poplar Hospital for Accidents. Including this, about £6,000 has been sent to Mr. Holland. This is a remarkable result in so short a time, for Mr. Sydney Holland's appeal is hardly ten days old. The appeal opened up questions of vital interest, and one useful object lesson is likely therefore to be overlooked. This is the valuable aid which a really energetic chairman may render the institution over which he presides. Too many chairmen are willing to accept the position of ornamental figure-heads or domestic managers rather than that of propelling motor. The position is seldom offered to any but those who possess influence, but responsibility in the office too frequently begins with the inscription of a name on reports and appeal circulars, and ends with the occasional presidency at a meeting. Fortunate, indeed, are such hospitals as Poplar, whose chairman identifies himself heart and soul with the interests of the charity to which he lends his name, and is willing to advocate in public all that concerns its welfare. This is very different from, and much more important work than that of presiding over the internal affairs of a hospital, necessary as this is. It is seldom that an institution is lucky enough to obtain so outspoken a champion as the present chairman of the Poplar Hospital. We hope that his example and success may inspire many imitators.

